# Posterior Reversible Encephalopathy Syndrome (PRES) and the Uncommon Sequela: Mesial Temporal Sclerosis

**DOI:** 10.7759/cureus.52380

**Published:** 2024-01-16

**Authors:** Abraham Mengstu, Mathew N Chakko, Blake Salisbury, Jibran Fateh

**Affiliations:** 1 Radiology, Ascension Providence Hospital / Michigan State University College of Human Medicine, Southfield, USA; 2 Neuroradiology, Ascension Providence Hospital / Michigan State University College of Human Medicine, Southfield, USA

**Keywords:** medically refractory epilepsy, mesial temporal lobe hyperintensities, mesial temporal lobe epilepsy, pres syndrome, neuroradiology, neuroimaging, posterior reversible encephalopathy syndrome (pres), hippocampal sclerosis, ammon’s horn sclerosis, mesial temporal sclerosis (mts)

## Abstract

Posterior reversible encephalopathy syndrome is often linked to conditions like hypertension and is characterized by reversible brain edema. The development of mesial temporal sclerosis as a consequence of posterior reversible encephalopathy syndrome is an uncommon clinical outcome.

We report a 48-year-old female who initially presented with severe iron deficiency anemia, hypertension, and septic tenosynovitis requiring surgical drainage with subsequent development of posterior reversible encephalopathy syndrome accompanied by endocarditis. Although there was a question of one seizure episode during one of her hospital days, the patient experienced multiple seizure episodes three months after she left the hospital. Subsequent MRI demonstrated atrophy of the left mesial temporal lobe suggesting mesial temporal sclerosis.

The temporal development of mesial temporal sclerosis in a patient with posterior reversible encephalopathy syndrome highlights mesial temporal sclerosis as a potential long-term consequence of posterior reversible encephalopathy syndrome, and the need for imaging surveillance in patients diagnosed with posterior reversible encephalopathy syndrome.

## Introduction

Posterior reversible encephalopathy syndrome (PRES) is reversible brain edema often associated with risk factors like hypertension, immunosuppressive therapies, eclampsia, and various other triggers [1,2]. The syndrome typically manifests with symptoms such as severe headaches, altered mental status, visual disturbances, seizures, and focal neurological deficits [1,2].

The diagnosis of PRES relies on a combination of clinical manifestations and neuroimaging results. The typical imaging feature of PRES is identification of bilateral subcortical white matter edema, characterized by hypodensities on non-contrast-enhanced computed tomographic (NCCT) images, and hyperintense lesions on T2 weighted spin-echo/Fluid-attenuated inversion recovery (FLAIR) magnetic resonance sequences, predominantly located in the posterior cerebral hemispheres [3]. Although the parieto-occipital lobes are most commonly involved in PRES (98%), frequencies ranging from 10%-68% have been reported for particular regions of the brain [2]. Different studies have also proposed the introduction of the term posterior reversible encephalopathy syndrome with spinal cord involvement (PRES-SCI) in a case series [4], with additional cases published elsewhere [5].

Mesial temporal sclerosis (MTS) as a direct consequence of PRES has been reported in a few pediatric cases, as detailed in the discussion of this report. We present a 48-year-old female patient who was diagnosed with PRES and developed MTS three months after the initial diagnosis. 

## Case presentation

A 48-year-old female presented to the hospital with complaints of pain and swelling of the left index finger of four days duration. Her past medical history was unremarkable except for headaches, anxiety, allergy to penicillin, obesity (BMI at presentation of 34 kg/m^2^), menorrhagia, and hypertension which the patient acknowledged on different subsequent inquiries. The patient denied fever, traumatic injury, insect bite, history of diabetes mellitus, and autoimmune or immunodeficiency diseases. Family and social history were non-contributory. Physical examination revealed tenderness and swelling, with an overall clinical impression of left index finger flexor tenosynovitis. Laboratory investigations showed severe iron deficiency anemia (hemoglobin of 4.6 - 5.1 g/dL), mild leukocytosis (WBC count 12,800 -13,300 cells/mcl), and mild thrombocytopenia (platelet count of 118,000 -125,000 cells/mcl). The hand radiograph was unremarkable for osteomyelitis. The patient was given a single dose of IV cefepime and switched to IV vancomycin. Subsequently, the patient underwent left index finger surgical debridement. Wound culture identified Methicillin-Sensitive Staphylococcus Aureus (MSSA). She was subsequently admitted to the floor, resumed on IV antibiotics, and transfused with four units of packed red blood cells over the course of 6 days.

Workup for the underlying etiology of the patient’s anemia with complete blood count and iron studies was compatible with iron deficiency anemia. Upper and lower gastrointestinal endoscopy were negative for gastrointestinal bleeding or malignancy. Contrast-enhanced CT scans of the abdomen/pelvis revealed no significant abnormalities except for the presence of a uterine fibroid that was concurred with subsequent pelvic ultrasound. The etiology of the iron deficiency anemia was attributed to the patient's menorrhagia.

**Figure 1:**
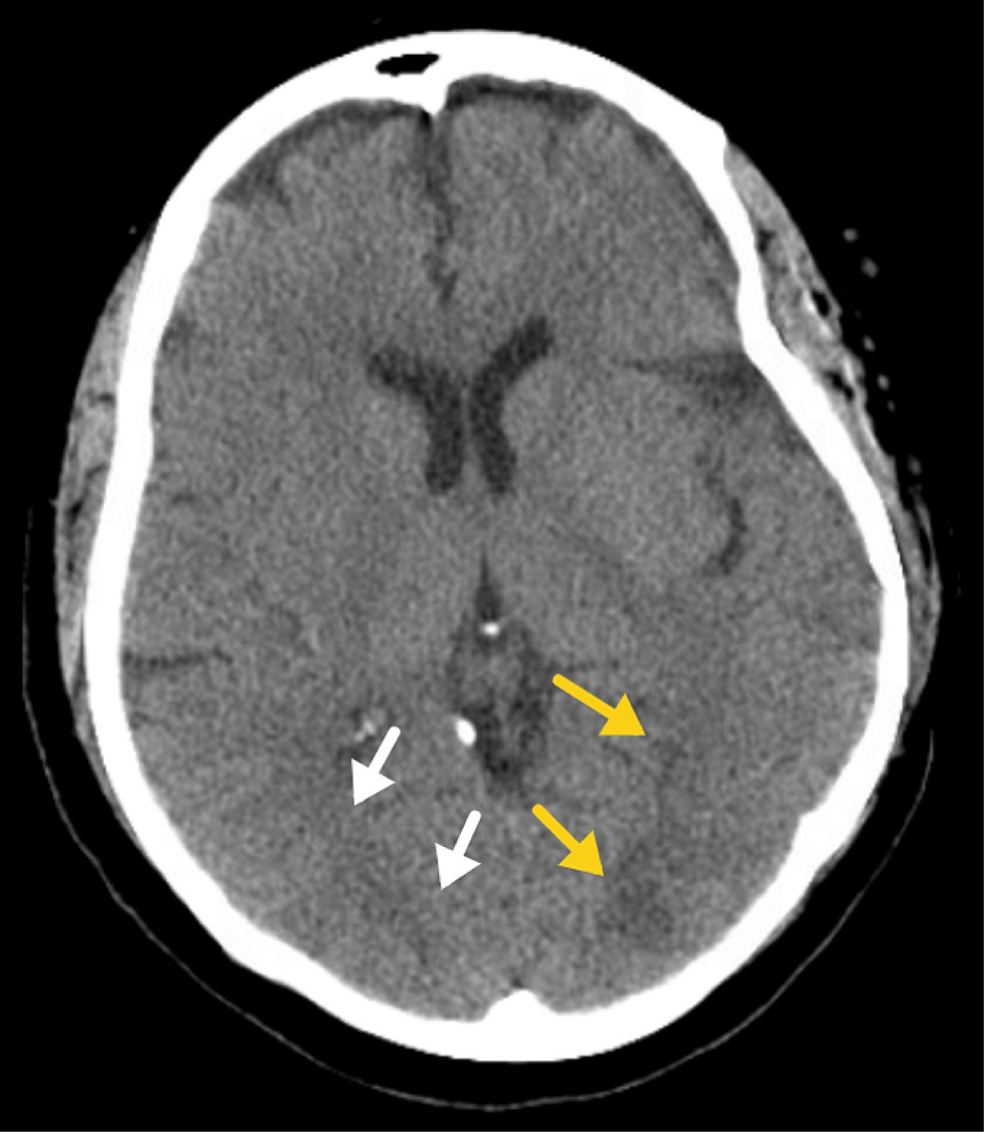
Representative axial computed tomographic images of the brain demonstrate hypodensities in the left (yellow arrows) greater than right (white arrows) parieto-occipital lobes raising the possibility of posterior reversible encephalopathy syndrome.

**Figure 2:**
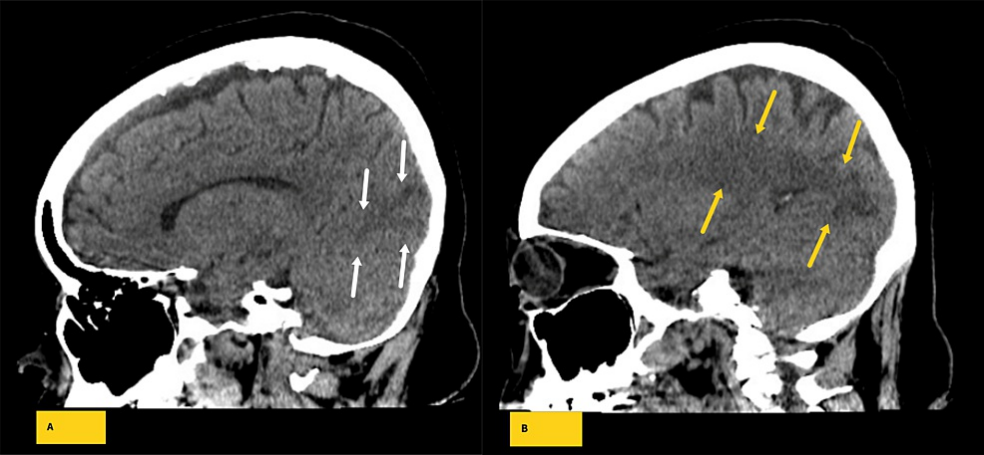
Representative sagittal computed tomographic images of the brain demonstrate hypodensities in the left (B - yellow arrows) greater than right (A - white arrows) parieto-occipital lobes raising the possibility of posterior reversible encephalopathy syndrome.

Starting on the day of her upper and lower gastrointestinal endoscopy and continuing for the next two days after the procedure, the patient experienced recurrent spikes in systolic blood pressure, with readings ranging from 150 to 193 mmHg. On the 3rd day after the endoscopy (7th day after the tenosynovectomy), the patient developed confusion (alert and oriented X 1). Part of the confusion might have been compounded by lorazepam given for known anxiety. Encephalopathy secondary to multiple factors, including sepsis from tenosynovitis, was considered, and a non-contrast CT scan of the head/brain was obtained. Subtle white matter hypodensities were seen in the bilateral occipital lobes with mild associated surrounding edema (Figure 1,2). A repeat physical exam obtained after the patient underwent the CT exam demonstrated mild improvement in mental status, with patient alert and oriented X2 and an NIH score of 1-3. 

The patient's blood pressure was managed initially with chlorthalidone 25 mg PO daily with multiple subsequent medication optimizations that included hydralazine 10 mg IV three times per day, a combination of chlorthalidone 25 mg PO daily and hydralazine 10 mg IV three times per day, and finally a combination of chlorthalidone 25 mg PO daily and hydralazine 15 mg IV three times per day. Her systolic blood pressure was maintained in the range of 117 to 164 mmHg. Of note, a few days of permissive hypertension were considered in the management of this patient, with considerations of possible multifocal septic/ischemic infarcts in the setting of infective endocarditis. The diastolic blood pressure remained within normal limits. 

**Figure 3:**
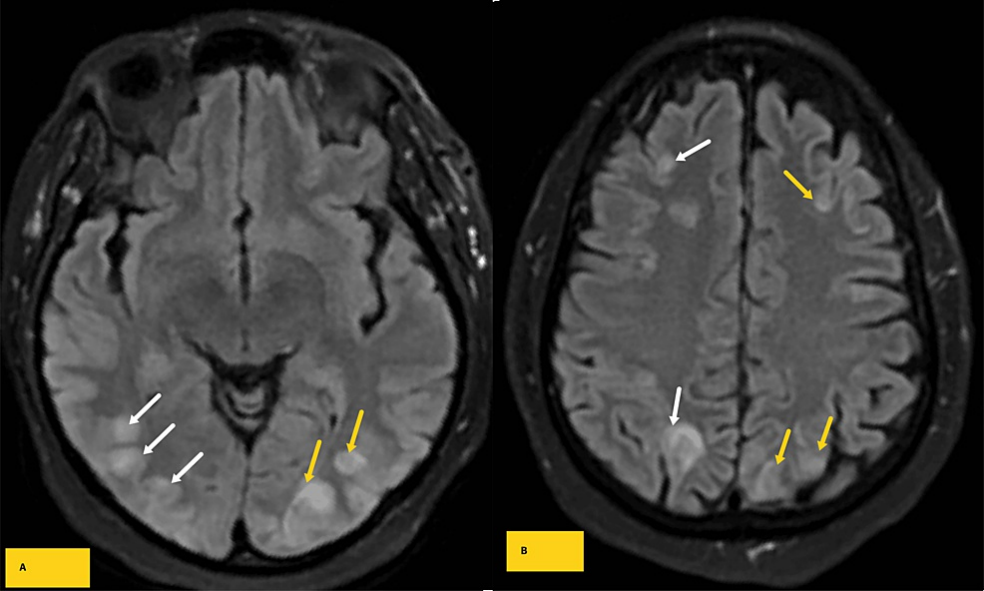
Axial fluid inversion recovery sequence (FLAIR) MR images through parieto-occipital lobes (A) and high frontoparietal (B) lobes extending to the posterior frontal lobes demonstrating multifocal subcortical areas of inversion recovery sequence (FLAIR) signal intensities in the parieto-occipital and high frontal subcortical areas corresponding to findings on the same day CT and compatible with posterior reversible encephalopathy syndrome.

A few hours after the onset of confusion, the patient's condition deteriorated again, with the patient becoming nonverbal. There was a question of focal tonic-clonic seizurse involving the left face and arm, and the patient was started on a 750 mg IV loading dose of Levetiracetam, followed by 500 mg IV Levetiracetam twice daily throughout her hospital stay. Subsequent brain MRI showed multifocal edema within the deep cortical and subcortical white matter of the bilateral occipital lobe distribution without diffusion restriction or magnetic susceptibility, with overall findings compatible with uncomplicated PRES (Figure 3).

Echocardiography revealed the presence of endocarditis with a 0.7 cm mitral valve vegetation. Subsequently, a repeat culture identified MSSA, a similar etiologic agent of the patient’s tenosynovitis. At this point, the patient's antibiotics were switched to IV Linezolid, which was continued during the patient's in-house stay and outpatient follow-up for a total of 6 weeks under the direction of infectious disease service.

The patient gradually improved with clear verbal communication and tolerating oral intake. However, residual visual, cognitive, and motor impairments necessitated placement in a subacute rehabilitation center. After arranging outpatient follow-up with hand surgery, infectious disease, cardiology, and neurology services, the patient was discharged to a rehabilitation center. 

While in the rehabilitation center, the patient continued to experience seizure episodes that necessitated admission to an outside facility. The seizures were controlled with Brivaracetam 100 mg twice daily, Lacosamide 100 mg twice daily, and Eslicarbazepine 400 mg daily, and the patient was discharged.

**Figure 4:**
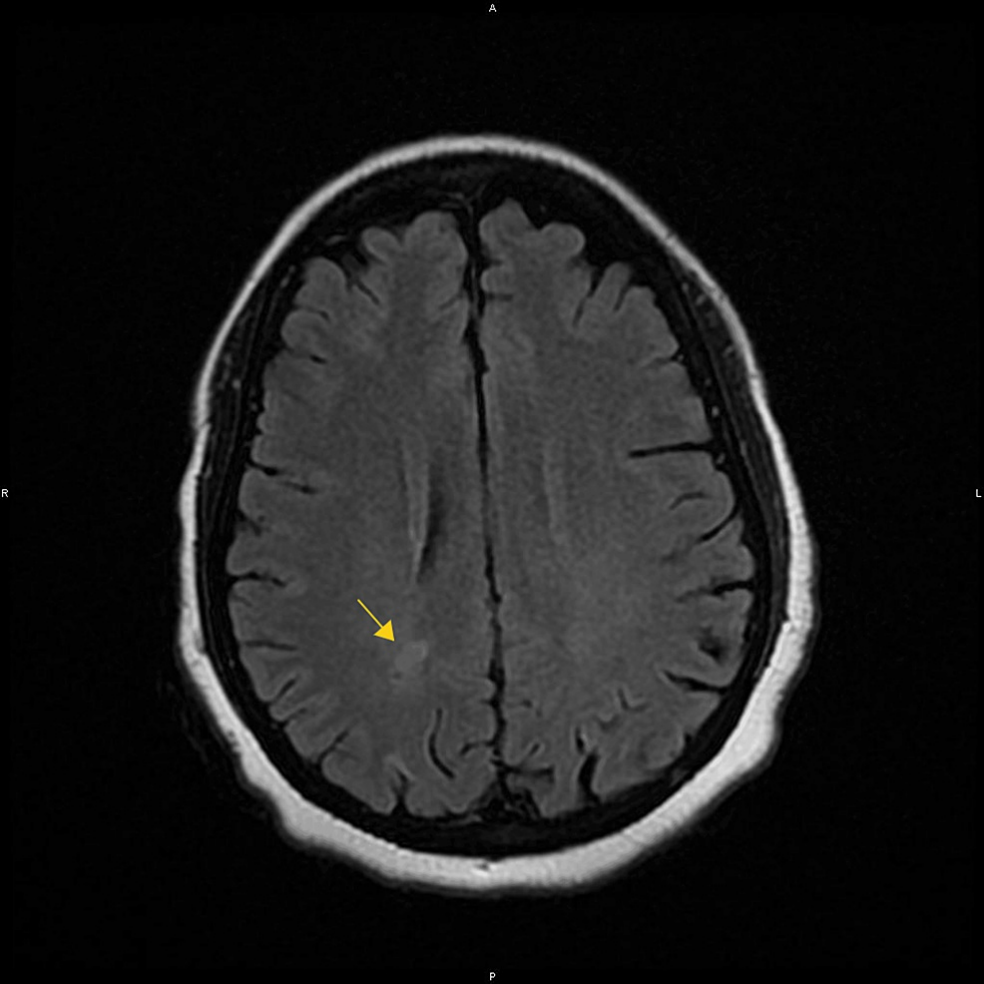
Axial FLAIR images demonstrate interval resolution of most subcortical parieto-occipital hyperintensities with minor residual Juxtacortical signal (yellow arrow) predominantly on the right parieto-occipital subcortical white matter compatible with the natural history of posterior reversible ischemic encephalopathy syndrome.

**Figure 5:**
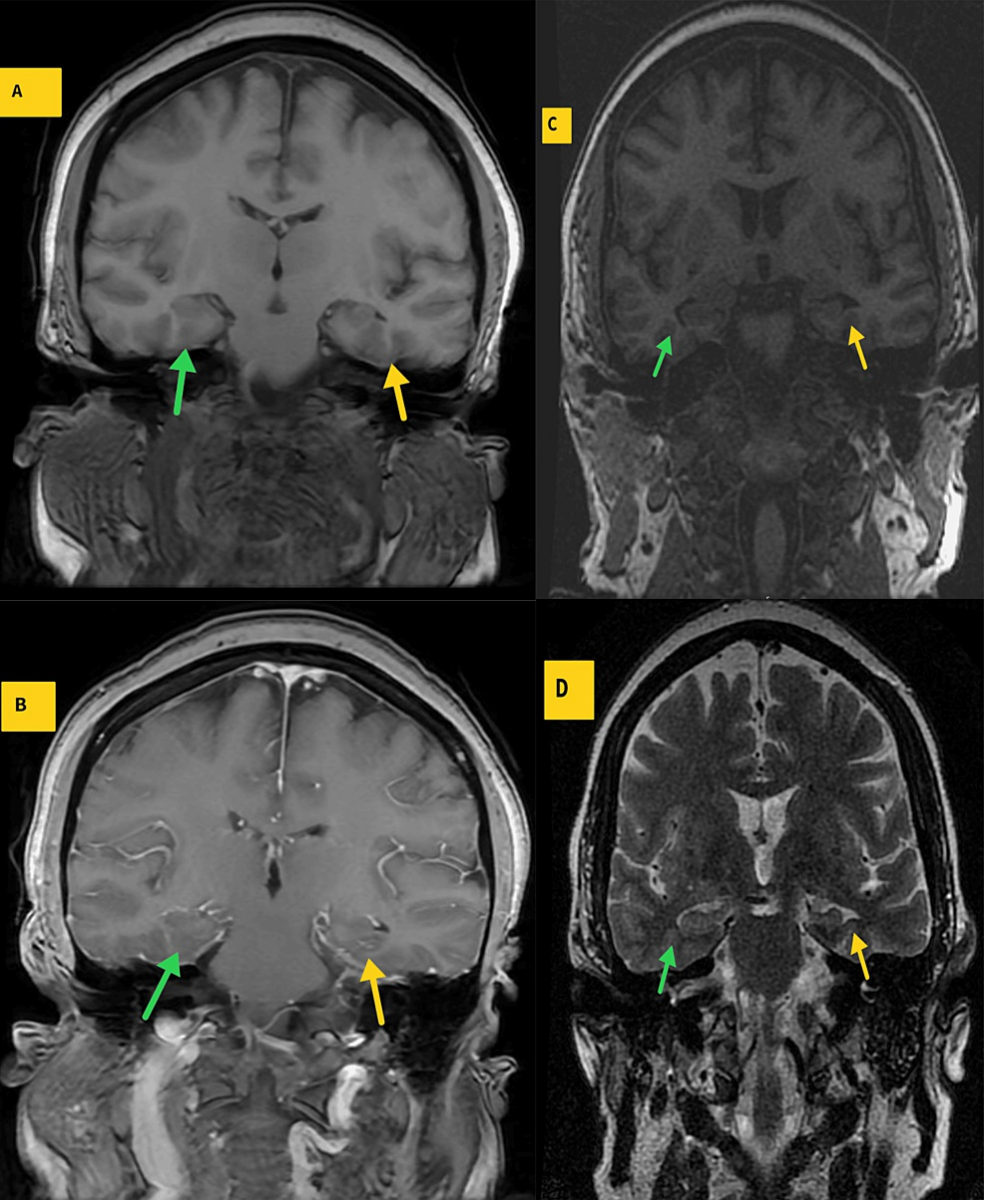
Representative coronal T1-weighted fast-spin echo (A) and post-contrast T1-weighted fast-spin echo (B) MR images through the temporal lobes during the posterior reversible encephalopathy syndrome (PRES) diagnosis demonstrate normal appearing right (A and B - green arrows) and left (A and B - yellow arrows) hippocampi without evidence of atrophy or enhancement. Representative coronal T1-weighted fast-spin echo (C) and T2-weighted fast-spin echo (D) 3 months after the diagnosis of PRES demonstrate asymmetric relative atrophy of the left mesial temporal lobe (C and D - yellow arrows) compared to the still normal appearing right temporal lobe (C and D - green arrows) with overall findings compatible with left mesial temporal sclerosis.

About three months after her initial discharge, a follow-up brain MRI obtained in the outpatient setting demonstrated interval resolution of most parieto-occipital fluid-attenuated inversion recovery (FLAIR) hyperintensities with minor residual juxtacortical FLAIR signal hyperintensities (Figure 4). Most striking was the temporal development of an apparent atrophy of the left medial temporal lobe that raised the question of MTS (Figure 5).

A few days after this brain MRI was obtained, the patient was sent to our facility by her neurologist for worsening headaches and right arm tremors. The patient continued to demonstrate residual bilateral vision impairment. The initial admission plan was to perform lumbar puncture for cerebrospinal fluid analysis and optimize anti-epileptic medications. Upon clinical evaluation, the patient continued to have persistent severe anemia and developed a small boil in the left breast with reactive left axillary lymph nodes. The left breast boil was opened and drained. The patient was treated with IV vancomycin and discharged with clindamycin. The patient was transfused with four units of packed RBCs, and the plan for lumbar puncture or neuroimaging was deffered given the lack of convincing clinical evidence for CNS infection. 

## Discussion

In this case discussion, we have outlined the complicated course of a 48-year-old female patient who initially presented for left index finger flexor tenosynovitis with subsequent development of PRES while under inpatient care. Subsequently, the patient developed seizures with MRI findings of left hippocampal volume loss compatible with mesial temporal sclerosis. 

The mesial (also medial) temporal lobe refers to the composite of the amygdala, hippocampus, parahippocampal gyrus, dentate gyrus, and uncus [6]. The hippocampus is the infolding gray matter of the medial temporal lobes with head, body, and tail parts. Due to a lack of consensus and ill definition of the hippocampus and adjacent cortex, the more general term hippocampal formation is mostly used to describe the dentate gyrus, including the fourth cornu ammonis (CA4), parahippocampal gyrus and hippocampus subfields (Cornu ammonis - CA1, CA2 and CA 3) [7]. 

A wide range of and debilitating clinical conditions affect the mesial temporal lobe [8]. MTS (also known as Ammon’s horn sclerosis, hippocampal sclerosis) refers to the histopathologic response to various insults and injuries characterized by loss of pyramidal neurons in the dentate gyrus, CA1, CA4, and to some extent CA3 subfields of the hippocampi, with associated gliosis [9]. Certain algorithms categorized hippocampal lesions (by implied translation mesial temporal diseases) into one of congenital, neurodegenerative, infectious/inflammatory, neoplastic, vascular, or toxic-metabolic diseases [10]. While these causes can initially affect a localized area, the interconnectedness of the limbic system might lead to the recruitment of the entire system in the pathogenesis of various disease entities [9].

PRES typically exhibits a reversible course, with significant patient recovery often observed upon addressing the underlying cause. However, prognosis can be influenced by factors such as the initial clinical severity, extent of brain edema, hyperglycemia, coagulopathy, and the presence of hemorrhage on imaging [11]. Fatal outcomes have been associated with hemorrhagic PRES as well as hypertension and neoplasm-related PRES [11,12]. 

Short to intermediate-term clinical outcomes of PRES include disease recurrence, particularly when underlying causes remain untreated or suboptimally controlled, progression into malignant PRES characterized by extensive edema and possible mass effect necessitating decompression craniotomy), development of seizure or epilepsy, motor deficits, and reported mortality rates of up to 3.2% to 19% [12,13]. Residual structural lesions were observed in up to 43% of patients diagnosed with PRES on follow-up imaging obtained 5 days to 12 months following initial diagnosis [14]. An autopsy conducted approximately 4.5 years after the resolution of clinical and radiological indications of PRES reported white matter rarefaction, isolated microinfarcts, subpial gliosis, and perivascular lymphocytic clusters with hemosiderin deposition in areas previously affected by PRES [15]. Although part of these findings may have been influenced by other underlying medical conditions, systemic lupus erythematosus (SLE) in this particular case, the authors raised questions regarding the truly "reversible" nature of this disease entity. 

This emerging evidence is challenging the long-characteristic concept of reversible brain edema associated with PRES. There have been few pediatric case reports of MTS that followed PRES. Aboian et. al documented a case of MTS in a pediatric patient who developed PRES subsequent to methotrexate chemotherapy for Burkitt lymphoma [16]. In a more recent study, it was reported that among hemato-oncology and stem cell transplant children, 9 out of 70 children diagnosed with PRES experienced the development of neurologic and imaging features of MTS [17]. In a separate case report, an 8-year-old girl, initially diagnosed with PRES, later developed pharmacotherapy-resistant epilepsy, with a follow-up MRI after 3 years revealing MTS [18]. 

The clinical presentation of MTS can vary, potentially leading to cognitive dysfunction, pharmacotherapy-resistant temporal lobe epilepsy, or functional impairment, contingent on the underlying cause [8,9]. The patient in this case report had residual cognitive decline that persisted after the first diagnosis of PRES, with multiple seizure episodes seen three months following the initial insult.

Although computed tomography and nuclear imaging can be helpful imaging diagnostic modalities, the diagnosis of MTS relies on magnetic resonance imaging (including 3-D volumetry and T2 weighted spin echo sequence relaxometry) findings. Magnetic resonance imaging (MRI) typically reveals T2 weighted spin echo signal hyperintensities within the affected mesial temporal lobe. However, T2 weighted spin echo signal hyperintensities are non-specific and can be seen with a wide range of clinical conditions. Pathology remains the definitive diagnostic modality, with characteristic findings of hippocampal atrophy, neuronal loss in the dentate gyrus, CA1, and CA3/CA4 hippocampal subfields and associated gliosis being typical features [19].

In an attempt to specify imaging differential diagnostic considerations, an algorithm by Alves et al. categorized hippocampal lesions into nodular (space-occupying) and non-nodular lesions [10] depending on whether these lesions lead to expansion or atrophy of the hippocampus respectively. The presence of associated hippocampal volume loss, which is indicative of neuronal loss, is a characteristic and relatively specific feature seen with MTS [20]. Besides volumetric indexes, conditions such as hippocampal ischemia, status epilepticus, low-grade tumors, and certain inflammatory/infectious encephalitis may exhibit peculiar features such as diffusion restriction and variable degrees of post-gadolinium enhancement patterns [10]. 

This particular patient demonstrated the temporal development of left mesial temporal lobe atrophy with associated subtle T2 signal hyperintensities (Figure 5) compatible with MTS. The temporal observation of these findings underscores the potential long-term consequence of PRES and the need for surveillance imaging in patients diagnosed with PRES. 

The management of MTS depends on the underlying etiology and may include disease-modifying therapies, seizure control, and rehabilitation with partial or complete amygdalohippocampectomy as the last management options for individuals with refractory epilepsy. 

Emerging evidence indicates that PRES may contribute to the development of chronic structural lesions, including MTS. However, the existing knowledge is primarily driven by case reports and retrospective analyses, limiting validity. The inherent constraints of case reports, the lack of temporal lobe epilepsy protocolled images such as 3D volumetry and fiber tracking acquisitions, as well as the absence of longitudinal imaging data in our specific case report impose similar limitations on the evidence presented. However, given the cumulative evidence and potential therapeutic implications for patients diagnosed with PRES, further investigation into the possible cause-and-effect relationships between PRES and MTS is recommended.

## Conclusions

In summary, we presented a 48-year-old female patient who was initially diagnosed with PRES, and temporal development of MTS on subsequent MRI obtained for new-onset seizures highlighting the possible cause-and-effect relationships between PRES and MTS.

Compelling data from case reports suggest that MTS could be a potential complication of PRES. While this emerging evidence requires further validation, it would be prudent to consider short- and intermediate-term follow-up imaging. MRI findings indicating hippocampal volume loss are consistent with the diagnosis of MTS. Implementing surveillance imaging enables early detection of MTS, facilitating early initiation of anti-epileptic medication and prevention of excitatory neurotoxicity associated with untreated or delayed epilepsy treatment. 
